# Effectiveness of Mobile Applications for Suicide Prevention: A Systematic Review and Meta-Analysis

**DOI:** 10.3390/bs15101345

**Published:** 2025-10-01

**Authors:** Kisun Sim, Sung-Man Bae

**Affiliations:** 1 Institute for Mental-Health, Dankook University, 119 Dandae-ro, Cheonan 31116, Republic of Korea; kisuns15@dankook.ac.kr; 2 Department of Psychology and Psychotherapy, College of Health Science, Dankook University, 119 Dandae-ro, Cheonan 31116, Republic of Korea

**Keywords:** mobile applications, suicide, mHealth, meta-analysis

## Abstract

Mobile applications are increasingly used for suicide prevention; however, their effectiveness remains unclear. This meta-analysis evaluated the effectiveness of mobile applications for suicide prevention and investigated potential moderators influencing intervention outcomes. Following the PRISMA guidelines, four databases (ProQuest, PubMed, Cochrane Central, and IEEE) were systematically searched for studies published from January 2020 to February 2025. This review was registered with PROSPERO (ID: CRD420251029046). Twenty-two studies were included, comprising 47 effect sizes derived from 6556 participants (3623 in the intervention and 2933 in the control groups). Risk of bias was assessed using RoB 2 (RCTs) and ROBINS-I (NRSs). Effect sizes were synthesized using random-effects meta-analysis with heterogeneity and publication bias evaluated. The overall post-intervention effect size was small to moderate (*g* = 0.39), with sustained but smaller effects observed at follow-up (*g* = 0.15). Moderator analyses indicated stronger effects for universal interventions targeting adults, weekly interventions, 12-week programs, and those implemented in efficacy settings. The findings should be interpreted with caution because of substantial heterogeneity. Nevertheless, the effects were statistically significant and provided evidence for the utility of mobile applications in suicide prevention, underscoring the need for further research to refine intervention design and delivery.

## 1. Introduction

Suicide is one of the most pressing and severe outcomes of mental health conditions, and remains a major global public health concern. According to the World Health Organization (World Health Organization, 2021), more than 720,000 individuals die by suicide each year, making suicide the third leading cause of death among individuals aged 15–29 years. Psychological interventions such as Cognitive Behavioral Therapy (CBT), Dialectical Behavior Therapy (DBT), and safety planning have been extensively studied over the past five decades and shown to be effective in reducing suicidal thoughts and behaviors (Fox et al., 2020; Rainbow et al., 2024).

Despite its efficacy, a large proportion of individuals who experience suicidal ideation, plans, and attempts do not have access to timely and adequate care. Estimates suggest that only 30–70% of individuals at risk of suicide seek or engage in mental health services (Hom et al., 2015; Torok et al., 2020). Barriers include a lack of trained providers, high treatment costs, stigma surrounding mental health, and limited access to traditional care (Priester et al., 2016; Knaak et al., 2017). These challenges highlight the need for accessible and scalable alternatives to bridge intervention gaps.

Owing to these barriers, digital mental health interventions (DMHIs) have received increasing attention. Among these, mobile applications represent one of the most prominent formats, with over 10,000 mental health applications currently available for download (Torous et al., 2018). The growth of DMHIs has been further accelerated by the COVID-19 pandemic, which has limited access to in-person services and heightened the demand for remote psychological support (Jacobson et al., 2020). Recent evidence indicates that digital interventions are effective in reducing symptoms of depression and anxiety, supporting their use as evidence-based alternatives to traditional psychotherapy (Moshe et al., 2021). Given their timeliness, anonymity, and cost-efficiency, mobile applications are increasingly recognized as scalable and sustainable solutions for improving access to mental healthcare across diverse populations.

With the rapid expansion of digital mental health technologies, interventions targeting suicidal ideation and its related symptoms have received increasing global attention (Cardoso et al., 2023; Oh et al., 2024). However, many mobile applications have been released without rigorous scientific evaluation, raising concerns about their safety and effectiveness. For instance, only six of the 20 applications reviewed by De la Torre et al. (2017) included evidence-based information, and most commercially available applications remain unvalidated. A recent systematic review emphasized that adverse events are infrequently and inconsistently reported in trials of mental health applications, leaving the potential for insufficiently evaluated harm (Linardon et al., 2024).

Several meta-analyses have assessed digital interventions in suicide prevention. Witt et al. (2017) reported that mobile applications may outperform waitlist controls post-intervention (*OR* = 0.36). However, this effect was not significant at follow-up, limiting the clinical implications of the findings. Torok et al. (2020) compared digital interventions targeting suicidality directly versus those addressing related symptoms (e.g., depression), and found that direct interventions significantly reduced suicidal ideation (*g* = 0.25), whereas indirect interventions failed to achieve statistical significance. However, the analysis included both web- and app-based interventions, thereby limiting our ability to isolate the unique effectiveness of mobile applications. Similarly, Oh et al. (2024) found moderate effects of digital psychotherapies for suicide prevention (*g* = 0.48) but reported that two-thirds of the included interventions were web-based, with only a small subset utilizing mobile applications. In contrast, Cardoso et al. (2023) reported no difference in suicidal ideation between mobile application interventions and control conditions, showing a small, nonsignificant effect in favor of the control condition.

Thus, the effectiveness of mobile applications in reducing suicidality remains inconclusive with previous findings showing inconsistent outcomes. Moreover, most meta-analyses were based on data collected before 2020, limiting their ability to reflect recent technological advances and emerging intervention strategies. The COVID-19 pandemic has led to the increased development and dissemination of mobile applications specifically targeting suicide prevention, which has been accompanied by a growing body of empirical studies evaluating their impact (Moshe et al., 2021; Oh et al., 2024). Synthesizing this updated evidence is critical, because establishing the efficacy of these novel approaches is an urgent global public health priority (Torok et al., 2020).

This meta-analysis aimed to evaluate the effectiveness of mobile applications designed to reduce suicide risk, focusing on studies published over the past five years. Specifically, this study examined whether such interventions lead to greater improvements in outcomes directly targeting suicidality (e.g., suicidal ideation, suicide attempts, and other suicide-related measures) and indirectly addressing suicide risk through depression and anxiety, compared with control conditions. To deepen our understanding of when and for whom these interventions are most effective, this review also explored the potential moderators across the four domains identified in previous research.

Participant characteristics included age group, recognizing that previous research examined mixed samples of adolescents and adults (Torok et al., 2020) and interventions focusing exclusively on children and adolescents (Conley et al., 2022), allowing for the investigation of potential developmental differences. The target population was categorized (Oh et al., 2024) as universal (general population), selected (individuals with identifiable risk factors for suicide but without current suicidal thoughts or behaviors), or indicated (individuals at high risk exhibiting suicidal thoughts or behaviors).

Intervention characteristics included the theoretical framework (e.g., CBT and third-wave approaches), treatment length, and session frequency. Previous research suggests that the effective dose of mobile mental health applications may differ, with improvements in mood and functioning observed as early as two weeks, the largest effects in some randomized controlled trials occurring at eight weeks, and user disengagement often beginning at four weeks (Comtois et al., 2022).

Methodological characteristics included study design (randomized controlled trial vs. nonrandomized study), type of control condition (treatment as usual, waitlist, attention placebo, and other mobile applications), and intervention setting (controlled research environments vs. real-world clinical contexts). Considering concerns regarding the ethical and practical challenges of conducting RCTs (Randomized Controlled Trials) with high-risk suicidal populations (Cardoso et al., 2023; Torok et al., 2020), both RCTs and nonrandomized studies with control group comparisons were included.

Finally, outcome type was classified into interventions directly targeting suicide-related variables (e.g., suicidal ideation and suicide attempts) and those indirectly addressing suicide risk through depression and anxiety. Torok et al. (2020) compared digital interventions directly targeting suicidality with those indirectly addressing suicide risk by targeting depression, reporting significant effects only for interventions focused on suicidality. Although their analysis was limited to depression, both depression and anxiety are well-established risk factors for suicidality, and evidence suggests that suicidal thoughts and behaviors frequently emerge in the context of depressive and anxiety symptoms (Barua et al., 2022). Based on this evidence, the present study examined depressive and anxiety symptoms as potential indirect pathways to suicidality.

By examining the contextual conditions and intervention-design features that influence outcomes, this review contributes to a more sophisticated understanding of digital approaches to suicide prevention and informs the development of evidence-based, personalized, and responsive interventions for individual needs across diverse settings.

## 2. Materials and Methods

This systematic review and meta-analysis adhered to the Preferred Reporting Items for Systematic Reviews and Meta-Analyses (PRISMA) guidelines (Page et al., 2021). This study was registered with PROSPERO (ID: CRD420251029046).

### 2.1. Search Strategy

The search focused on articles published between January 2020 and February 2025 to exclude outdated methods. To ensure a comprehensive review across multiple disciplines, four electronic databases were searched: Psychology (ProQuest), Medicine (PubMed), Clinical Trials (Cochrane Central Register of Controlled Trials), and Engineering and Technology (IEEE Xplore). All database searches were conducted on 17 March 2025.

Search terms were selected based on three key components: target population, intervention type, and study design. To identify relevant studies, a combination of terms related to suicide (“suicide” OR “suicidal*”), mobile application (“app” OR “application” OR “mHealth” OR “mobile” OR “phone”), and control groups (“control” OR “controlled” OR “clinical trial”) was utilized.

### 2.2. Eligibility Criteria

The eligibility criteria for this study were as follows:

- Populations: Studies targeting individuals with suicidal thoughts, plans, or prior suicide attempts without age restrictions.
- Intervention: Studies were eligible if mobile applications explicitly featured suicide-related components, even when only depression or anxiety outcomes were assessed using standardized instruments, or if suicide-related outcomes were measured regardless of whether the application explicitly targeted suicide prevention. Studies were excluded if the application did not include suicide-related content or assessed suicide-related outcomes. Interventions limited to brief text messaging or email reminders were also excluded. When suicide was assessed using a single item from an existing instrument (e.g., Item 9 of PHQ-9), the study was included, but the single-item measure was excluded from the effect size calculations.
- Comparator: Studies that include a comparison group (e.g., psychoeducation, waiting list).
- Outcomes: Outcomes of interest were classified as (1) direct measures of suicidality (e.g., suicidal ideation and suicide attempts) and (2) indirect measures of suicide risk through depression and anxiety. Only studies that assessed these outcomes using self-report instruments with established psychometric reliability and validity were included. The instruments used in each study are listed in Table S1.
- Study design: Studies using randomized or nonrandomized controlled designs.
- Language: Studies published in English.
- Date range: Studies published between January 2020 and February 2025.
- Availability: Full-text articles.

### 2.3. Article Selection

The literature search and article selection processes involved reviewing the titles and abstracts of articles retrieved through database searches. The eligibility of the studies was assessed by two independent raters (K. S. and S. L). Any disagreements were resolved through discussion with the corresponding author. A PRISMA flow diagram provides an overview of the number of studies excluded during the screening process, along with the reasons for exclusion.

### 2.4. Risk of Bias and Methodological Quality

The results of individual studies are potentially susceptible to various sources of bias, which may lead to either an overestimation or underestimation of the true effect of the intervention. To account for this, a systematic risk of bias assessment was conducted using instruments recommended by the Cochrane Collaboration. For randomized controlled trials (RCTs), the Risk of Bias 2 (RoB 2) tool was used, whereas for nonrandomized studies (NRSs), the Risk of Bias in Nonrandomized Studies of Interventions (ROBINS-I) tool was applied.

The RoB 2 tool evaluates five domains of potential bias: randomization process, deviations from intended interventions, missing outcome data, outcome measurement, and selection of the reported result. Each domain was rated as having low, unclear, or high risk of bias. The ROBINS-I tool comprises seven domains: confounding, classification of interventions, study selection, deviations from intended interventions, missing data, outcome measurement, and selection of reported results. Each domain was categorized as having a low, moderate, or critical risk of bias.

### 2.5. Data Extraction

Two independent reviewers (K. S. and S. L.) extracted the data using a standardized spreadsheet, recording participant characteristics (age and sex), intervention details (application name, treatment length, session frequency, follow-up periods), methodological features (study design and type of control condition), and study characteristics (publication year and country), and reported the outcomes. Outcomes of interest comprised direct measures of suicidality (e.g., suicidal ideation, suicide attempts, and other suicide-related indicators) and indirect measures reflecting suicide risk limited to depression and anxiety. These two variables were included as indirect outcomes because they are well-established risk factors for suicidality, and were consistently assessed across the included studies. The theoretical framework of each intervention was also coded based on the descriptions provided in the included studies, with the absence of an explicit framework recorded as “not reported”. All analyses were conducted using the total scores from the validated psychometric instruments. For each outcome domain, all reported results across different measures and time points were extracted without selective restrictions.

For each planned synthesis, study characteristics were systematically tabulated and mapped onto the predefined categories for subgroup and moderator analyses (e.g., age group, intervention type, treatment length, session frequency, control condition, and study setting). Studies were included in the synthesis if they reported outcome data relevant to the planned comparisons, and all available results across measures and time points were extracted without selective restriction.

In most studies, pre- and post-intervention means and standard deviations for the experimental and control groups were available and converted into Hedges’ g for analysis. In two studies (Bisconti et al., 2024; Josifovski et al., 2024), only ANOVA results were reported; therefore, the F values were transformed into Hedges’ g. All effect size conversions were performed using Comprehensive Meta-Analysis (CMA) version 4.0. For Goldstein et al. (2025), subgroup means and standard deviations were not reported; thus, the corresponding author was contacted and the necessary data were obtained directly. Individual study characteristics and effect sizes are tabulated in the summary tables, including details of participants, interventions, comparators, outcomes, and study design.

### 2.6. Statistical Analysis

All statistical analyses were conducted using CMA 4.0 and R (version 4.5.0), with the “meta” and “metafor” packages employed to compute and synthesize effect sizes (Viechtbauer, 2010). The standardized mean difference (Hedges’ *g*) was used as the primary effect size metric.

A random-effects model was used to account for expected heterogeneity among the studies. Heterogeneity was assessed using Cochran’s *Q* statistic and Higgins’ *I*^2^ index, with *I*^2^ values greater than 75% indicating substantial heterogeneity (Higgins et al., 2003). Forest plots were generated to visually inspect effect size dispersion, and sensitivity analyses were conducted to evaluate whether any single study influenced the pooled effect estimates.

Effect sizes were calculated based on Hedges’ *g*, which estimates the standardized mean difference in continuous outcomes between the intervention and control conditions while correcting for small-sample bias. Standardized effect sizes were interpreted following Cohen’s (1988) guidelines, with values of 0.20, 0.50, and 0.80, representing small, moderate, and large effects, respectively.

To address the nonindependence of effect sizes, special consideration was given to studies that reported multiple effect sizes derived from the same sample. Including multiple effect sizes from a single study can artificially inflate precision and bias of pooled estimates. To mitigate this risk, we adopted Cooper’s (1998) shifting unit of analysis approach. Specifically, independent effect sizes were computed at the study level for primary analyses, whereas individual effect sizes were retained for subgroup analyses. This approach preserves statistical independence without discarding valuable information, thereby ensuring analytical rigor and data completeness.

## 3. Results

### 3.1. Selection and Inclusion of Studies

**Figure 1:**
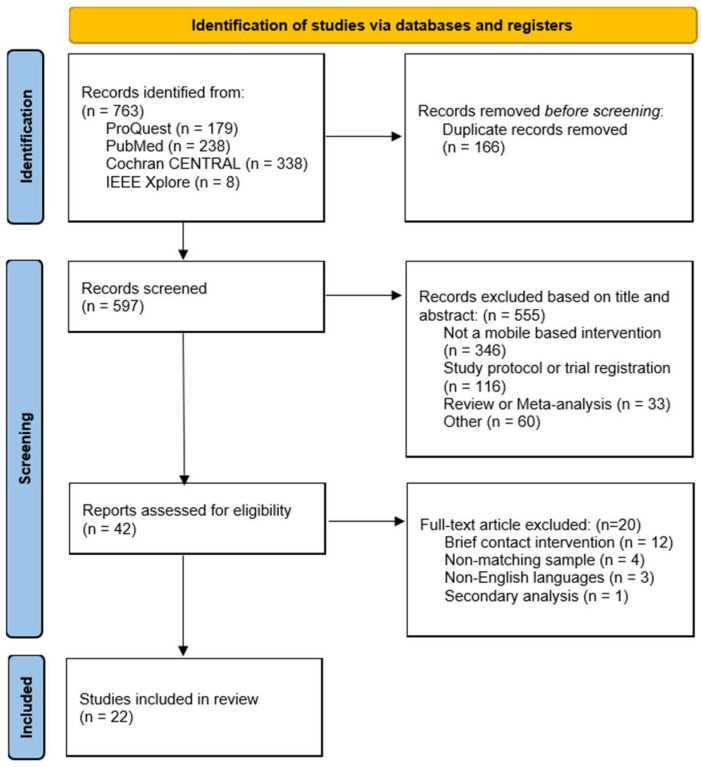
PRISMA flow diagram of study selection process.

In total, 597 articles were identified through the database searches after removing duplicates (Figure 1). After screening of titles and abstracts, 555 articles were excluded based on the predefined eligibility criteria. Of the 42 articles extracted for full-text review, 20 were excluded for the following reasons: 12 involved brief contact interventions (e.g., SMS or telephone check-ins) rather than structured mobile applications, four targeted stakeholders or caregivers (e.g., parents) instead of individuals at risk for suicide, and three were published in non-English languages. Additionally, although McGillivray et al. (2023) met all inclusion criteria, they were excluded because they constituted a secondary analysis of data already included in Torok et al. (2022), and the inclusion of both studies would have resulted in duplicate data, violating the assumption of independence.

In total, 22 articles met the inclusion criteria and were included in the final meta-analysis (Supplementary Materials S1). These articles collectively included 6556 participants: 3623 in the intervention group and 2933 in the control group. Some studies (e.g., Comtois et al., 2022; Horwitz et al., 2024) included multiple mobile applications, resulting in 25 distinct intervention groups. In total, 47 effect sizes were coded for analysis owing to the inclusion of multiple outcome variables and intervention arms.

### 3.2. Characteristics of the Included Studies

The characteristics of the 22 included studies are summarized in Table S1, which provides detailed information on the intervention features. Table 1 presents a descriptive overview of study level and participant characteristics. In terms of publication year, most studies were published in 2024 (k = 9, 40.9%), followed by 2022 (k = 6, 27.3%), 2021 (k = 3, 13.6%), and early 2025 (k = 2, 9.1%). One study each (4.5%) was published in 2020 and 2023. Most studies were conducted in the United States (k = 9, 40.9%), followed by Australia (k = 3, 13.6%). One study each (4.5%) was conducted in the following ten other countries: Argentina, China, Denmark, Iran, Japan, Kazakhstan, Poland, South Korea, Taiwan, and the United Kingdom.

Most samples were drawn from adult populations (k = 16, 72.7%), with six studies (27.3%) targeting adolescents. The mean age of all the studies was 25.8 years (SD = 9.5). Women comprised the majority of the participants, accounting for 75.3% of the total sample. Most studies employed RCTs designs (k = 21, 95.5%), with only one (4.5%) using a nonrandomized design.

The intervention duration ranged from a single 2 h session to a year. The most commonly reported intervention durations were 4 and 12 weeks, each reported in seven studies (31.8%), followed by 6 weeks in three studies (13.6%).

Follow-up assessments were reported in 11 studies (50.0%). The most commonly reported duration was 12 weeks (k = 3, 13.6%), followed by 16 weeks (k = 2, 9.1%), 24 weeks (k = 2, 9.1%), and one year (k = 2, 9.1%). One study each (4.5%) reported follow-ups at 4 and 8 weeks.

### 3.3. Assessment of Study Validity

#### 3.3.1. Risk of Bias Assessment

A risk of bias assessment was conducted for all included studies using the tools recommended by the Cochrane Collaboration. The Risk of Bias 2 (RoB 2) tool was applied to 21 RCTs, and one nonrandomized study was assessed using the Risk of Bias in Nonrandomized Studies of Interventions (ROBINS-I).

**Figure 2:**
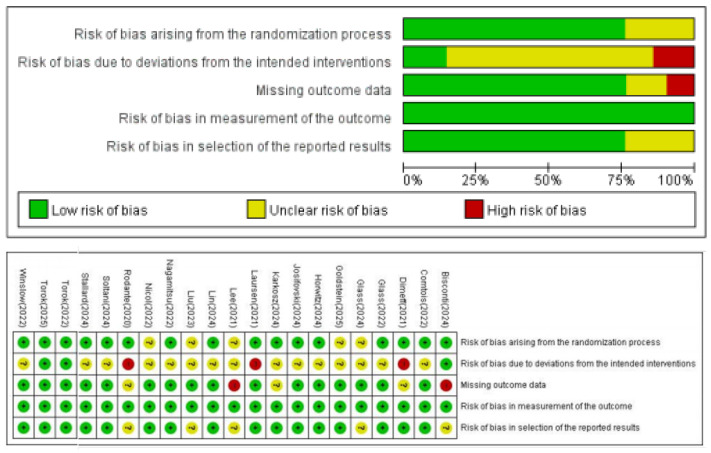
Risk of Bias (RoB) of the RCT studies. “+” = low risk of bias; “?” = unclear risk of bias; “-” = high risk of bias.

Among the 21 RCTs, five were rated as high risk of bias, 14 as some concern, and two as low risk. The most frequently observed source of bias was related to deviations from the intended interventions, primarily because of the lack of blinding of participants and researchers, a limitation that is often inherent in suicide prevention research. Additionally, two studies were rated as high risk because of missing outcome data exceeding 20%. A summary of the overall risk of bias ratings, as well as domain-specific ratings for each study, is presented in Figure 2.

A single nonrandomized study was rated as having a moderate overall risk of bias, primarily because of insufficient information regarding the classification of interventions and deviations from intended interventions.

#### 3.3.2. Outlier Analyses

**Figure 3:**
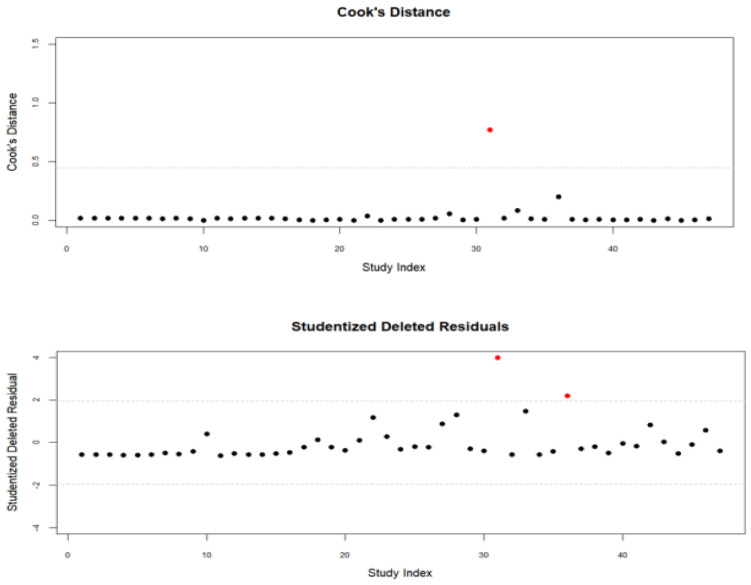
Scatterplots showing the distribution of the values for Cook’s Distance and Studentized Deleted Residuals. Red dots = outliers; Black dots = included studies.

Outliers were examined using studentized deleted residuals and Cook’s distance. Effect sizes with absolute residuals greater than ±1.96 were identified as statistical outliers, resulting in detection of two such cases (Figure 3). Among these, one effect size exceeded the Cook’s D threshold of 0.45, indicating a potential influence on the pooled estimates. Following Viechtbauer and Cheung (2010), a leave-one-out sensitivity analysis was conducted to assess the robustness of the overall effect size. Based on this analysis, one effect size (anxiety; Soltani et al., 2024) that substantially affected the pooled estimate was excluded from subsequent analyses to improve the robustness of the findings.

#### 3.3.3. Publication Bias

**Figure 4:**
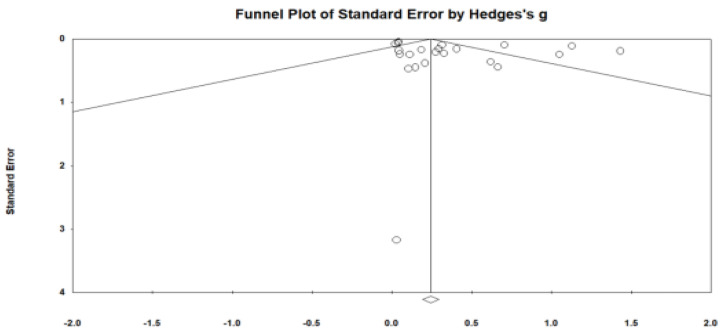
Funnel Plot assessing publication bias in the included studies.

Publication bias was assessed using three methods: Begg and Mazumdar’s rank correlation test, Duval and Tweedie’s trim-and-fill procedure, and the classic fail-safe N method (Figure 4). Begg’s test yielded a nonsignificant result (Kendall’s *τ* = 0.07, *p* = 0.63), indicating no statistical evidence of funnel plot asymmetry. The trim-and-fill analysis did not impute any missing studies and the adjusted effect size remained identical to the observed estimate, suggesting that the pooled effect was not meaningfully influenced by potential missing studies. Furthermore, the classic fail-safe N indicated that 572 unpublished null-effect studies are required to nullify the observed significance, supporting the robustness of the findings.

### 3.4. Main Effects of Mobile Applications

#### 3.4.1. Primary Effects

**Figure 5:**
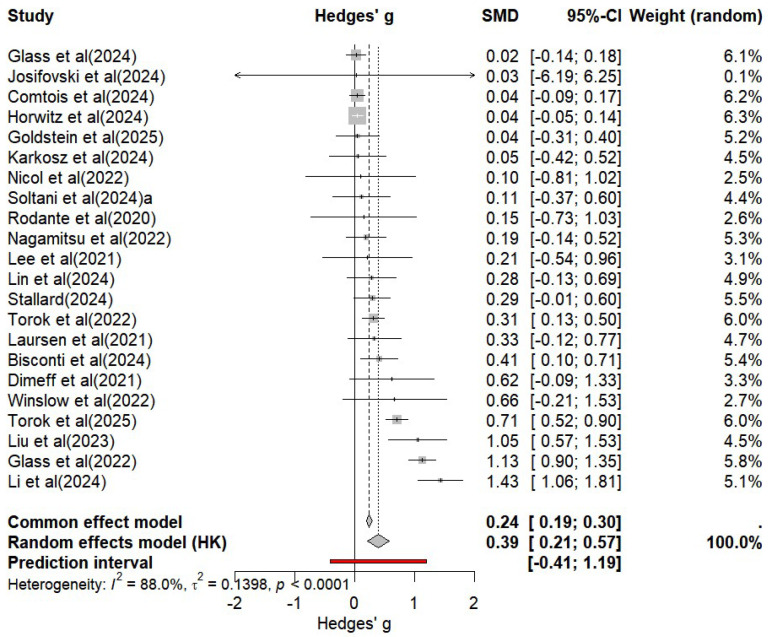
Forest Plot of the overall effectiveness of mobile applications on suicidality.

The analysis of primary effects revealed substantial heterogeneity across studies evaluating mobile application interventions targeting suicide prevention (*I*^2^ = 88.0%, *p* < 0.001). Therefore, a random-effects model was used to determine the effect sizes. The pooled effect size was small to moderate, with a Hedges’ g of 0.39 (95% *CI* [0.21, 0.57], *p* < 0.01). A forest plot illustrating the effect sizes and confidence intervals of the included studies is shown in Figure 5.

The 95% prediction interval ranged from –0.38 to 1.16, suggesting considerable heterogeneity in the true effects that might be expected in future studies (Borenstein et al., 2009). Given that the primary objective of this meta-analysis was to characterize the overall effectiveness of digital interventions for suicide prevention, further analyses were conducted to examine potential moderators that may account for the variability in effect sizes across studies.

#### 3.4.2. Follow-Up Effects

Eleven studies reported follow-up effects, with assessment periods ranging from four months to one year. One study (Nicol et al., 2022) did not provide sufficient statistical information and was excluded. Another study (Torok et al., 2025) reported an unusually large effect size (*g* = 3.35), which was identified as a statistical outlier.

A meta-analysis of the remaining nine (k = 9) was conducted to estimate the effects of mobile app-based interventions at follow-up. The analysis yielded a statistically significant effect size of *g* = 0.15 (*SE* = 0.05, 95% *CI* [0.06, 0.24]), suggesting that mobile app-based interventions had a small but reliable impact on suicide-related outcomes at follow-up.

### 3.5. Moderator Analysis

#### 3.5.1. Participant Characteristics

Subgroup analyses were conducted to examine whether effect sizes (Hedges’ *g*) varied according to participant characteristics, specifically, age and target population type (Table 2). With respect to age group, no significant differences were observed between studies targeting adolescents (k = 6, *g* = 0.36, 95% *CI* [−0.22, 0.93]) and those targeting adults (k = 19, *g* = 0.33, 95% *CI* [0.16, 0.50]); the between-group difference was nonsignificant (*Q* (1) = 0.01, *p* = 0.92).

In contrast, significant differences were found across target population (*Q* (2) = 7.20, *p* = 0.02). Interventions targeting universal populations showed a moderate and statistically significant effect (k = 6, *g* = 0.46, 95% *CI* [0.04, 0.88]), whereas those targeting selected (k = 10, *g* = 0.36, 95% *CI* [−0.01, 0.74]) or indicated populations (k = 9, *g* = 0.09, 95% *CI* [−0.01, 0.18]) yielded nonsignificant effects.

Further interaction analysis between age group and target population revealed significant differences (*Q* (4) = 11.19, *p* = 0.02). The largest effect was observed in interventions targeting adult-universal populations (k = 4, *g* = 0.64, 95% *CI* [0.06, 1.23]). In contrast, interventions targeting adult-selected (k = 6, *g* = 0.20, 95% *CI* [−0.06, 0.45]), adolescent-indicated (k = 3, *g* = 0.19, 95% *CI* [−0.18, 0.55]), adult-indicated (k = 6, *g* = 0.07, 95% *CI* [−0.05, 0.20]), and adolescent-universal populations (k = 2, *g* = 0.05, 95% *CI* [−0.76, 0.87]) yielded small and statistically nonsignificant effects. The adolescent-selected subgroup was excluded from the analysis because it had only one effect size (k = 1), which did not allow for valid estimations or comparisons.

These findings suggest that the effectiveness of mobile applications for suicide prevention may differ depending on both the age group and risk level of the target population, with greater effects observed in universal prevention efforts among adults.

#### 3.5.2. Intervention Characteristics

Subgroup analyses were conducted to examine whether intervention characteristics, specifically the theoretical framework, session frequency, and treatment length, moderated the effectiveness of mobile applications for suicide prevention (Table 3).

Regarding the theoretical framework, no significant differences in effect sizes were observed across the subgroups (*Q* (3) = 0.20, *p* = 0.98). Although the between-group difference was not statistically significant, interventions based on CBT (k = 10, *g* = 0.30, 95% *CI* [0.12, 0.48]) and third-wave approaches (k = 4, *g* = 0.33, 95% *CI* [0.19, 0.47]) showed small-to-moderate effects. In contrast, interventions based on other frameworks (k = 4, *g* = 0.32, 95% *CI* [−0.44, 1.08]) and those without a reported theoretical orientation (k = 7, *g* = 0.40, 95% *CI* [−0.16, 0.96]) yielded nonsignificant results, as their confidence intervals included zero.

In terms of session frequency, significant differences were observed (*Q* (2) = 11.90, *p* = 0.01). Interventions delivered on a weekly basis demonstrated the largest effect (k = 8, *g* = 0.58, 95% *CI* [0.18, 1.00]) compared to those delivered as needed (k = 10, *g* = 0.27, 95% *CI* [−0.01, 0.54]) or on a daily basis (k = 6, *g* = 0.07, 95% *CI* [−0.04, 0.18]). One study that used a single-session intervention was excluded from the analysis because of insufficient data for valid estimation (k = 1).

Significant variation was also found across treatment lengths (*Q* (6) = 27.42, *p* < 0.00). Interventions lasting 12 weeks yielded the largest effect size (k = 7, *g* = 0.61, 95% *CI* [0.11, 1.11]). In contrast, interventions lasting 8 weeks (k = 4, *g* = 0.80, 95% *CI* [−1.17, 2.27]), six weeks (k = 4, *g* = 0.12, 95% *CI* [−0.11, 0.35]), and 4 weeks (k = 8, *g* = 0.10, 95% *CI* [−0.01, 0.21]) yielded nonsignificant results, as their confidence intervals included zero. Studies with treatment durations of 2, 24, and 48 weeks were excluded because each study contributed only to a single effect size (k = 1), preventing a reliable subgroup estimation.

These findings suggest that both the frequency and duration of mobile app-based interventions may moderate their effectiveness.

#### 3.5.3. Methodological Characteristics

Subgroup analyses were performed to examine whether methodological characteristics, specifically, the type of control condition and study setting, moderated the effectiveness of mobile device applications in suicide prevention (Table 4).

Regarding the type of control condition, no statistically significant differences in effect sizes were observed across subgroups (*Q* (3) = 4.00, *p* = 0.26). Interventions compared with a waitlist controls demonstrated the largest effect size (k = 6, *g* = 0.66, 95% *CI* [0.07, 1.26]), followed by treatment-as-usual (TAU) conditions (k = 8, *g* = 0.25, 95% *CI* [0.11, 0.39]). In contrast, interventions compared to attention placebo controls (k = 4, *g* = 0.37, 95% *CI* [−0.50, 1.25]) and placebo application conditions (k = 7, *g* = 0.18, 95% *CI* [−0.06, 0.41]) yield nonsignificant effects, as their confidence intervals included zero.

Regarding study setting, a significant moderating effect was identified (*Q* (1) = 6.86, *p* = 0.01). Studies conducted in efficacy settings (e.g., controlled research environments) showed a moderate and statistically significant effect (k = 16, *g* = 0.41, 95% *CI* [0.16, 0.65]), whereas those conducted in effectiveness settings (e.g., real-world clinical contexts) reported a smaller and nonsignificant effect (k = 9, *g* = 0.09, 95% *CI* [−0.01, 0.18]).

These findings suggest that the effectiveness of mobile interventions for suicide prevention may be reduced in real-world clinical settings, underscoring the importance of ecological validity and implementation context in evaluating digital mental health interventions.

#### 3.5.4. Outcome Type

To examine whether intervention effects varied according to the outcome type, subgroup analyses were conducted to compare direct (i.e., suicidality) and indirect outcomes (i.e., depression and anxiety) (Table 5). The difference in effect sizes across these outcome types was not statistically significant (*Q* (2) = 2.28, *p* = 0.32). These findings suggest that mobile interventions may be broadly applicable for addressing both suicide risk and comorbid emotional distress, such as depression and anxiety.

## 4. Discussion

This study aimed to comprehensively evaluate the current evidence base for mobile applications designed for suicide prevention and identify key factors that may moderate intervention outcomes. In total, 22 studies published between January 2020 and February 2025 were included in the meta-analysis, comprising 6556 participants. By synthesizing evidence on the effectiveness of mobile applications, this study aimed to assess their potential to deliver accessible and impactful interventions, particularly for individuals who face barriers to engage in traditional mental health services. The main findings of this meta-analysis are summarized as follows.

### 4.1. Summary of Main Findings

First, the mobile application interventions were effective in reducing suicide-related outcomes, with statistically significant effects compared to control conditions. The pooled effect size was small to moderate (*g* = 0.39, 95% *CI* [0.21, 0.57]), with statistically significant effects observed at both post-intervention and follow-up assessments (*g* = 0.15, 95% *CI* [0.06, 0.24]). This overall effect size was comparable to that of traditional psychological interventions for suicide attempts, as reported in previous meta-analyses (*g* = 0.45; Hofstra et al., 2020). These findings indicate that mobile applications may offer effectiveness comparable to that of traditional face-to-face interventions. Considering the limited availability and accessibility of traditional mental health services, mobile interventions can serve as meaningful supplements. Specifically, they may enhance both access to care and continuity of support for at-risk individuals who may otherwise remain underserved.

Second, subgroup analysis based on participant characteristics showed that a large proportion of the included studies focused on adults (k = 19), whereas only a small number targeted children and adolescents (k = 6). No significant differences in effect size were observed between the two age groups. When categorized by intervention target level, interventions targeting universal populations (*g* = 0.46) demonstrated significantly larger effect sizes than those targeting selected (*g* = 0.36) or indicated (*g* = 0.09) populations, suggesting that mobile applications may be more effective preventive tools for the general population than targeted interventions for individuals with elevated or high suicide risk. This pattern differs from the findings of Chen and Chan (2022), who reported larger effects for children and adolescents (*d* = 0.29) than for young adults (*d* = 0.13) or adults (*d* = 0.19), although both studies showed the strongest effects for universal interventions (*d* = 0.26). Notably, this meta-analysis combined age and target variables, and revealed that universal interventions targeting adults yielded the largest effect size (*g* = 0.64). These results suggest that mobile applications may be particularly effective as a preventive tool when delivered to the general adult population rather than exclusively to high-risk individuals.

Third, subgroup analyses based on intervention characteristics revealed no statistically significant differences in effect sizes across the theoretical frameworks, contrary to prior assumptions. However, interventions without a clear theoretical basis or categorized as “other” (e.g., motivational interviewing) did not yield significant effects. In contrast, interventions grounded in CBT or third-wave approaches (e.g., DBT and ACT) were associated with statistically meaningful results. Although previous research has suggested that third-wave therapies may outperform CBT in suicide prevention contexts (Torok et al., 2020), further research is required to identify the most appropriate theoretical models for delivery via mobile applications. Interventions delivered weekly (*g* = 0.58) were more effective than those delivered daily or on demand. This finding suggests that mobile applications incorporating structured weekly therapeutic elements may be more effective. Furthermore, interventions lasting 12 weeks (*g* = 0.61) achieved greater effects than shorter programs, consistent with previous findings (Graham et al., 2020). These results highlight the importance of employing an evidence-based theoretical framework and optimal delivery format for designing mobile interventions for suicide prevention.

Fourth, subgroup analyses based on methodological characteristics revealed no significant differences in effect sizes according to the type of control condition, suggesting the robustness of mobile device interventions for suicide prevention. Although both randomized and nonrandomized controlled trials were included in the present meta-analysis, most studies employed randomized designs, limiting the ability to detect differences in effect sizes based on the study design. Most studies were conducted in experimental contexts (efficacy, k = 16), yielding a moderate effect size (*g* = 0.41), whereas those conducted in real-world clinical settings (effectiveness, k = 9) showed negligible effects (*g* = 0.09). These findings are consistent with those of Moshe et al. (2021), who reported a higher effect size in efficacy trials (*g* = 0.59) than in effectiveness trials (*g* = 0.30), attributing this difference to variations in treatment adherence. Only six studies in the present review reported adherence data, limiting further analysis. Future studies should systematically examine the role of adherence in shaping intervention outcomes.

Finally, this study compared the effectiveness of mobile interventions that directly targeted suicide outcomes with those that indirectly addressed suicidality by focusing on depression or anxiety as primary outcomes. The results showed no statistically significant differences in the effect sizes based on the type of outcome targeted, contrasting with previous findings that reported stronger effects for interventions specifically targeting suicidality (Torok et al., 2020). These findings suggest that mobile interventions may be applicable across multiple mental health domains, potentially operating through shared therapeutic mechanisms regardless of the primary outcome targeted. Further research is needed to elucidate these mechanisms and determine strategies for optimizing digital interventions for individuals at risk of suicide.

### 4.2. Clinical Considerations

This meta-analysis offers valuable insights into the clinical application of mobile application interventions for suicide prevention. The largest effects were found in universal interventions targeting adults, indicating that such tools may be more effective for broad preventive use than for individuals experiencing acute crises. These findings highlight the need for careful identification of user risk levels to ensure that those with elevated or imminent suicide risk are appropriately referred for intensive clinician-supported services. As previous research suggests, digital interventions should be adapted to an individual’s level of suicide risk, whether characterized by suicidal thoughts, planning, or previous attempts (Jha et al., 2023), and should incorporate appropriate safety plans for high-risk users.

Further research is required to clarify the mechanisms by which mobile app-based interventions exert their effects. Consistent with psychotherapy research that distinguishes between common and specific therapeutic factors (Wampold, 2007), future studies should identify the components of mobile applications that actively contribute to change as well as the psychological processes underlying their impact (Song & Bae, 2022). However, most of the included studies provided limited details regarding the type and modality of the intervention. In particular, it was often unclear whether the applications were designed for fully self-directed use, delivered as adjuncts to face-to-face treatment, or integrated into structured clinical approaches such as safety planning (Rainbow et al., 2024; Ferguson et al., 2022). Similarly, specific intervention approaches have rarely been described in sufficient detail, restricting our ability to offer more clinically tailored recommendations. This lack of reporting constrains our ability to draw firm conclusions regarding clinical applicability. Addressing both the mechanisms of change and specific modalities of application use will be critical for advancing the development of more targeted and clinically effective interventions.

Finally, a successful implementation requires further empirical validation. Incorporating qualitative feedback from end users and practitioners is essential for evaluating the usability, acceptability, and contextual appropriateness of mobile applications. Such insights can support the integration of digital tools into existing mental health services and promote sustainability in real-world practice (Abascal-Peiró et al., 2024). In addition, this meta-analysis did not address risks or adverse events as safety-related outcomes were not reported in the included studies, highlighting the need for future research to systematically evaluate the safety of mobile applications for suicide prevention.

### 4.3. Limitations

Despite the contributions of this meta-analysis, several limitations should be considered when interpreting its findings. First, although the overall effect size was statistically significant, substantial heterogeneity across studies (*I*^2^ = 88%) indicated considerable variability in intervention effects. Moreover, the prediction interval (*PIs*) ranged widely from −0.38 to 1.16, suggesting that the true effect in future studies may vary from potentially negative to strongly positive. Prediction intervals are particularly informative in meta-analyses because they reflect the expected distribution of effects in future studies and provide a more realistic assessment of uncertainty (Borenstein et al., 2009). Thus, the wide interval underscores the need for cautious interpretation and highlights the value of prediction intervals in evaluating the robustness and generalizability of the findings.

Second, the moderator analyses yielded 95% confidence intervals that included zero, suggesting that the evidence for differential intervention effects across subgroups remains inconclusive. Given the limited number of studies within certain categories and their methodological heterogeneity, the current findings should be interpreted with caution. These limitations underscore the need for additional high-quality research with adequately powered subgroup analyses to more definitively determine the conditions under which intervention effects may vary.

Third, the generalizability of the findings is limited. Most of the included studies were conducted on adult populations; however, evidence regarding children, adolescents, and older adults is insufficient, even though suicide remains a critical concern in these groups. This gap underscores the need for further research, particularly when considering the potential challenges related to mobile device application accessibility in these populations. In addition, most studies have been conducted in Western contexts (e.g., the United States and Australia), highlighting the importance of future research that considers cultural and contextual variability. Furthermore, no studies included in this meta-analysis used death by suicide as an outcome measure; therefore, conclusions cannot be drawn regarding whether mobile applications prevent suicide mortality. Additionally, this meta-analysis only focused on applications developed since 2020, although several mobile applications created before 2020 remain in use. Re-examining these earlier applications is essential to provide a more comprehensive understanding of their effectiveness in real-world settings.

Finally, this review was unable to assess the role of treatment adherence as few studies have reported relevant data. Given that digital interventions frequently face challenges such as low engagement and high dropout rates, particularly in suicide prevention contexts, understanding how adherence influences outcomes is critical (Witt et al., 2017). In the present review, only a few studies (k = 6) provided adherence data, which precluded quantitative synthesis. Moreover, the required level of participant engagement was often unclear. For example, some interventions involve structured daily activities, whereas others relied on infrequent notifications. This lack of clarity limits our ability to interpret adherence data and evaluate how different levels of engagement affect intervention effectiveness. Future trials should incorporate standardized reporting of both adherence metrics and engagement protocols to clarify the relationships between usage patterns, intervention effectiveness, and long-term outcomes.

## 5. Conclusions

This meta-analysis provides evidence supporting the potential of mobile applications as effective tools for suicide prevention. Mobile app-based interventions have demonstrated significant effects across a range of populations and delivery formats, indicating their potential applicability beyond specific clinical settings. These findings suggest that mobile interventions can serve as valuable adjuncts to traditional mental health services by enhancing accessibility, continuity, and scalability of care. Although the significance of these results is clear, certain limitations must be acknowledged when interpreting them. Specifically, the findings cannot be generalized to older adults or populations who may face difficulties accessing mobile applications, underscoring the need for further research that specifically addresses these groups. As digital mental health technologies continue to advance, refining intervention content, elucidating the underlying mechanisms of change, and promoting effective integration into comprehensive suicide prevention strategies are crucial. Continued research and thoughtful implementation are essential to maximize the public health impact of these emerging digital tools.

## Figures and Tables

**Table 1:** Descriptive Summary of the Characteristics of the Included Studies (k = 22).

Name	Total
Number of studies	22
Publication year	
2020	1 (4.5%)
2021	3 (13.6%)
2022	6 (27.3%)
2023	1 (4.5%)
2024	9 (40.9%)
2025	2 (9.1%)
Country	
USA	9 (40.9%)
Australia	3 (13.6%)
Other	10 (45.5%)
Participant characteristics	
Age	*M* = 25.8, *SD* = 9.5
Child and Adolescents (k = 6, 27.3%)	*M* = 15.3, *SD* = 0.8
Adults (k = 16, 72.7%)	*M* = 28.7, *SD* = 8.6
Female	75.3%
Study design	
RCTs	22 (95.5%)
NRS	1 (4.5%)
Follow-up assessments	11 (50.0%)
4 weeks	1 (4.5%)
8 weeks	1 (4.5%)
12 weeks	3 (13.6%)
16 weeks	2 (9.1%)
24 weeks	2 (9.1%)
1 year	2 (9.1%)

Notes. RCTs = Randomized Controlled Trial; NRS = Nonrandomized Study.

**Table 2:** Moderator Analysis by Participant Characteristics.

Categories	Subgroup	k	*ES*	95% *CI*		*Q*	*df*	*p*
				Lower	Upper			
Age group	Adolescent	6	0.36	−0.22	0.93	0.01	1	0.92
	Adult	19	0.33	0.6	0.50			
Target population	Universal	6	0.46	0.04	0.88	7.20 *	2	0.02
	Selected	10	0.36	−0.01	0.74			
	Indicated	9	0.09	−0.01	0.18			
Age group _Target population	Adult_Universal	4	0.64	0.06	1.23	11.19 *	4	0.02
	Adult_Selected	9	0.20	−0.06	0.45			
	Adolescent _Indicated	3	0.19	−0.18	0.55			
	Adult_Indicated	6	0.07	−0.05	0.20			
	Adolescent _Universal	2	0.05	−0.76	0.87			

Notes. k = The number of *ES*; *ES* = Hedge’s *g*; *CI* = confidence interval; *df* = degree of freedom; *Q* = Q test heterogeneity; * = statistically significant.

**Table 3:** Moderator Analysis by Intervention Characteristics.

Categories	Subgroup	k	*ES*	95% *CI*		*Q*	*df*	*p*
				Lower	Upper			
Theorical framework	CBT	10	0.30	0.12	0.48	0.20	3	0.98
	3th wave	4	0.33	0.19	0.47			
	Other	4	0.32	−0.44	1.08			
	Not reported	7	0.40	−0.16	0.96			
Session frequency	Weekly	8	0.58	0.18	1.00	11.9 **	3	0.01
	Whenever needed	10	0.27	−0.01	0.54			
	Daily	6	0.07	−0.04	0.18			
Treatment Length	4 weeks	8	0.10	−0.01	0.21	27.42 ***	6	0.00
	6 weeks	4	0.12	−0.11	0.35			
	8 weeks	4	0.80	−1.17	2.27			
	12 weeks	7	0.61	0.11	1.11			

Notes. k = The number of *ES*; *ES* = Hedge’s *g*; *CI* = confidence interval; *df* = degree of freedom; *Q* = Q test heterogeneity; ** *p* < 0.01; *** *p* < 0.001.

**Table 4:** Moderator Analysis by Methodological Characteristics.

Categories	Subgroup	k	*ES*	95% *CI*		*Q*	*df*	*p*
				Lower	Upper			
Control Condition	Waitinglist	6	0.66	0.07	1.26	4.0	3	0.26
	TAU	8	0.25	0.11	0.39			
	Attention placebo	4	0.37	−0.50	1.25			
	Attention placebo application	7	0.18	−0.06	0.41			
Setting	Efficacy	16	0.41	0.16	0.65	6.86 **	1	0.01
	Effectiveness	9	0.09	−0.01	0.18			

Notes. k = The number of *ES*; *ES* = Hedge’s *g*; *CI* = confidence interval; *df* = degree of freedom; *Q* = Q test heterogeneity; ** *p* < 0.01.

**Table 5:** Moderator Analysis by Outcome Type.

Categories	Subgroup	k	*ES*	95% *CI*		*Q*	*df*	*p*
				Lower	Upper			
Direct outcome	Suicidality	16	0.18	0.05	0.32	2.28	2	0.32
Indirect outcome	Depression	18	0.37	0.15	0.59			
	Anxiety	12	0.22	0.02	0.41			

Notes. k = The number of *ES*; *ES* = Hedge’s *g*; *CI* = confidence interval; *df* = degree of freedom; *Q* = Q test heterogeneity.

## Data Availability

The data supporting the conclusions of this article will be made available by the authors upon request.

## Supplementary Materials

The following supporting information can be downloaded at: https://www.mdpi.com/article/10.3390/bs15101345/s1, S1: Final list of 22 studies included in the meta-analysis, Table S1: Detailed Characteristics of the included studies, Table S2: PRISMA Checklist.
