# Supplementary material for: Effectiveness of Mobile Applications for Suicide Prevention: A Systematic Review and Meta-Analysis
Source: Behav Sci (Basel). 2025 Oct 1;15(10):1345. doi: 10.3390/bs15101345 (PMC12561950; doi:10.3390/bs15101345)
Supplement: Supplementary file 1 [file behavsci-15-01345-s001.zip › S1. Final list of 22 studies included in the meta-analysis.pdf]

Supplementary Materials S1. Final List of 22 studies included in the Meta-Analysis

Bisconti, N., Odier, M., Becker, M., & Bullock, K. (2024). Feasibility and Acceptability of a Mobile App–Based TEAM-CBT (Testing Empathy Assessment Methods–Cognitive Behavioral Therapy) Intervention (Feeling Good) for Depression: Secondary Data Analysis. *JMIR Mental Health, 11*, e52369. https://doi.org/10.2196/52369

Comtois, K. A., Mata-Greve, F., Johnson, M., Pullmann, M. D., Mosser, B., & Arean, P. (2022). Effectiveness of Mental Health Apps for Distress During COVID-19 in US Unemployed and Essential Workers: Remote Pragmatic Randomized Clinical Trial. *JMIR mHealth and uHealth, 10*(11), e41689. https://doi.org/https://doi.org/10.2196/41689

Dimeff, L. A., Jobes, D. A., Koerner, K., Kako, N., Topher, J., Kelley-Brimer, A., Boudreaux, E. D., Beadnell, B., Goering, P., Witterholt, S., Melin, G., Samike, V., & Schak, K. M. (2021). Using a Tablet-Based App to Deliver Evidence-Based Practices for Suicidal Patients in the Emergency Department: Pilot Randomized Controlled Trial. *JMIR mental health, 8*(3), e23022. https://doi.org/https://doi.org/10.2196/23022

Glass, N., Bloom, T., Alexander, K. A., Emezue, C., Olawole, W., Clough, A., Turner, R., & Perrin, N. (2024). Effectiveness of the myPlan Teen App, a Digital Healthy Relationship and Safety Planning Intervention with Adolescent Aged 15-17 Years. *Journal of Adolescent Health, 75*(4), 665-672. https://doi.org/10.1016/j.jadohealth.2024.06.014

Glass, N. E., Clough, A., Messing, J. T., Bloom, T., Brown, M. L., Eden, K. B., Campbell, J. C., Gielen, A., Laughon, K., Grace Karen, T., Turner, R. M., Alvarez, C., Case, J., Barnes-Hoyt, J., Alhusen, J., Hanson, G. C., & Perrin, N. A. (2022). Longitudinal Impact of the myPlan App on Health and Safety Among College Women Experiencing Partner Violence. *Journal of interpersonal violence, 37*(13-14), NP11436-NP11459. https://doi.org/10.1177/0886260521991880

Goldstein, T. R., Kennard, B. D., Porta, G., Miller, A. O., Aguilar, K., Bigley, K., Vaughn-Coaxum, R. A., McMakin, D. L., Douaihy, A., Iyengar, S., Biernesser, C. L., Zelazny, J. & Brent, D. A. (2025). Bridging Gaps in Care Following Hospitalization for Suicidal Adolescents: as Safe As Possible (ASAP) and BRITE App. *Journal of the American Academy of Child and Adolescent Psychiatry, 64*(5), 612-624. https://doi.org/10.1016/j.jaac.2024.06.008

Horwitz, A. G., Mills, E. D., Sen, S., & Bohnert, A. S. B. (2024). Comparative Effectiveness of Three Digital Interventions for Adults Seeking Psychiatric Services: a Randomized Clinical Trial. *JAMA network open, 7*(7), e2422115. https://doi.org/10.1001/jamanetworkopen.2024.22115

Josifovski, N., Torok, M., Batterham, P., Wong, Q., Beames, J. R., Theobald, A., Holland, S., Huckvale, K., Riley, J., Cockayne, N., Christensen, H., & Larsen, M. (2024). Efficacy of BrighterSide, a Self-Guided App for Suicidal Ideation: Randomized Controlled Trial. *JMIR mental health, 11*, e.55528. https://doi.org/10.2196/55528

Karkosz, S., Szymański, R., Sanna, K., & Michałowski, J. (2024). Effectiveness of a Web-based and Mobile Therapy Chatbot on Anxiety and Depressive Symptoms in Subclinical Young Adults: Randomized Controlled Trial. *JMIR Formative Research, 8*, e47960. https://doi.org/10.2196/47960

Laursen, S. L., Helweg-Jørgensen, S., Langergaard, A., Søndergaard, J., Sørensen, S. S., Mathiasen, K., Lichtenstein, M. B., & Ehlers, L. H. (2021). Mobile Diary App Versus Paper-Based Diary Cards for Patients with Borderline Personality Disorder: Economic Evaluation. *Journal of Medical Internet Research, 23*(11), e28874. https://doi.org/10.2196/28874

Lee, M. R., & Cha, C. (2021). A Mobile Healing Program Using Virtual Reality for Sexual Violence Survivors: a Randomized Controlled Pilot Study. *Worldviews on evidence-based nursing, 18*(1), 50-59. https://doi.org/10.1111/wvn.12478

Li, A., Aimaganbetova, O., Baktybayev, Z., Koishibayeva, M., Makhmutov, A., & Murzagulova, M. (2024). Using modern technologies for prevention of suicidal behavior among adolescents. *Social Work in Mental Health, 22*(1), 73-90. https://doi.org/https://doi.org/10.1080/15332985.2023.2247514

Lin, Y. H., Wu, C. Y., Gau, B. S., Lin, C. H., Ho, H. Y., & Lou, M. F. (2024). Effectiveness Study of a Cultural Adaptation of Cognitive-Behavioural Therapy-Based Application for Depressive Symptoms in College Students: a Randomised Controlled Trial. *Journal of psychiatric and mental health nursing, 32*(3), 712-722. https://doi.org/10.1111/jpm.13146

Liu, C., Chen, H., Zhang, A., Gong, X., Wu, K., Liu, C.-Y., & Chiou, W.-K. (2023). The effects of short video app-guided loving-kindness meditation on college students' mindfulness, self-compassion, positive psychological capital, and suicide ideation. *Psicologia, Reflexão e Crítica, 36*(1), 32. https://doi.org/https://doi.org/10.1186/s41155-023-00276-w

Nagamitsu, S., Kanie, A., Sakashita, K., Sakuta, R., Okada, A., Matsuura, K., Ito, M., Katayanagi, A., Katayama, T., Otani, R., & et al. (2022). Adolescent Health Promotion Interventions Using Well-Care Visits and a Smartphone Cognitive Behavioral Therapy App: randomized Controlled Trial. *JMIR mHealth and uHealth, 10*(5), e34154. https://doi.org/10.2196/34154

Nicol, G., Wang, R., Graham, S., Dodd, S., & Garbutt, J. (2022). Chatbot-Delivered Cognitive Behavioral Therapy in Adolescents with Depression and Anxiety During the COVID-19 Pandemic: Feasibility and Acceptability Study. *JMIR Formative Research, 6*(11), e40242. http://doi.org/10.2196/40242

Rodante, D. E., Kaplan, M. I., Fedi, R. O., Gagliesi, P., Pascali, A., Quintero, P. S., Compte, E. J., Perez, A. I., Weinstein, M., Chiapella, L. C., & Daray, F. M. (2020) CALMA, a Mobile Health Application, as an Accessory to Therapy for Reduction of Suicidal and Non-Suicidal Self-Injured Behaviors: A Pilot Cluster Randomized Controlled Trial. *Archives of Suicide Research, 26*(2), 801-818. http://doi.org/10.1080/13811118.2020.1834476

Soltani, Z., Parizad, N., Radfar, M., Alinejad, V., Arzanlo, M., & Haghighi, M. (2024). The effect of the Yara smartphone application on anxiety, sleep quality, and suicidal thoughts in patients with major depressive disorder in Iran: a randomized controlled trial. *BMC psychiatry, 24*, 1-12. https://doi.org/https://doi.org/10.1186/s12888-024-05688-1

Stallard, P., Whittle, K., Moore, E., Medina-Lara, A., Morrish, N., Cliffe, B., Rhodes, S., & Taylor, G. (2024). Clinical effectiveness and safety of adding a self-harm prevention app (BlueIce) to specialist mental health care for adolescents who repeatedly self-harm: a single blind randomised controlled trial (the BASH study). *Psychiatry research, 339*, 116017. https://doi.org/10.1016/j.psychres.2024.116017

Torok M, Han J, McGillivray L, Wong Q, Werner-Seidler A, O'Dea B, et al. (2022) The effect of a therapeutic smartphone application on suicidal ideation in young adults: Findings from a randomized controlled trial in Australia. *PLoS Medicine, 19*(5), e1003978. https://doi.org/10.1371/journal.pmed.1003978

Torok, M., McGillivray, L., Gan, D. Z. Q., Han, J., Hetrick, S., & Wong, Q. J. J. (2025). Adherence and efficacy outcomes in young Australians with suicidal ideation using a self-management app and digital engagement strategy compared with a sham app: a three-arm randomised controlled trial. *EClinicalMedicine, 79*, 102963. https://doi.org/10.1016/j.eclinm.2024.102963

Winslow BD, Kwasinski R, Hullfish J, Ruble M, Lynch A, Rogers T, Nofziger D, Brim W & Woodworth C (2022). Automated stress detection using mobile application and wearable sensors improves symptoms of mental health disorders in military personnel. *Frontier in Digital Health, 4*, 919626. https://doi.org/10.3389/fdgth.2022.919626
