# Supplementary material for: Effectiveness of Mobile Applications for Suicide Prevention: A Systematic Review and Meta-Analysis
Source: Behav Sci (Basel). 2025 Oct 1;15(10):1345. doi: 10.3390/bs15101345 (PMC12561950; doi:10.3390/bs15101345)
Supplement: Supplementary file 1 [file behavsci-15-01345-s001.zip › Table S1. Detailed Characteristics of the included studies.pdf]

Table S1. Detailed Characteristics of the included studies

n	Author / Country	Study Design	Participants M(SD)	Name of the apps	Theorical Framework	Treatment Length	Session Frequency	Control condition	Outcome measure	Follo-up periods
1	Bisconti (2024) / USA	RCT	Adult 44.7 (12.4)	Feeling Good	CBT	4 weeks	Daily	waitinglist	PHQ-9 (Depression) GAD-7 (Anxiety)	-
2	Comtois (2022) / USA	RCT	Adult 31.1 (9.5)	COVID Coach / Calm / 7 Cups of Tea	N/R	4 weeks	Whenever needed	attention control app	SBQ-R (suicidal behaviors) PHQ-9 (Depression) GAD-7 (Anxiety)	-
3	Dimeff (2021) / USA	RCT	Adult 34.4 (15.2)	Jaspr Health	3th wave (DBT)	2 hours	Single session	Treatment as usual	SRCS (suicide coping)	-
4	Glass (2022) / USA	RCT	Adult 20.9 (2.3)	myPlan	N/R	24 weeks	Whenever needed	attention placebo	CESD-R (Depression)	1 year
5	Glass (2024) / USA	RCT	Adolescent 16.2 (0.8)	myPlan Teen	N/R	12 weeks	Whenever needed	attention placebo	PROMIS (Depression)	1 year
6	Goldstein (2025) / USA	RCT	Adolescent 14.6 (1.6)	BRITE	OTHER (MI)	4 weeks	Daily	Treatment as usual	C-SSRS (suicidal behaviors)	24 weeks
7	Horwitz (2024) / USA	RCT	Adult 36.8 (14.3)	Silvercloud / Headspace	CBT /OTHER (MINDFUL NEES)	6 weeks	Daily	attention control app	PANSI (suicidal behaviors) PHQ-9 (Depression) GAD-7 (Anxiety)	-
8	Josifovski (2024) / Australia	RCT	Adult 39.1 (13.5)	BrighterSid e	CBT	6 weeks	Whenever needed	waitinglist	SIDAS (suicidal ideation)	12 weeks
9	Karkosz (2024) / Poland	RCT	Adult 25.7 (4.5)	Fido	CBT	2 weeks	Whenever needed	attention placebo	CESD-R (Depression) PHQ-9 (Depression) STAI (Anxiety)	4 Weeks
10	Laursen (2021) / Denmark	RCT	Adult -	mDiary	3th wave (DBT)	1 year	Weekly	Treatment as usual	SBQ-R (suicidal behaviors) PHQ-9 (Depression)	-
11	Lee (2021)/ South Korea	RCT	Adult 21.9 (1.4)	Sister, I will tell you	Other (Mindulness)	4 weeks	Weekly (twice a week)	attention placebo	SIQ (suicidal ideation)	8 weeks
12	Li (2024) / Kazakhstan	NRS	Adolescent -	Happify	N/R	12 weeks	Weekly (twice a week)	waitinglist	BDI-II (Depression)	-
13	Lin	RCT	Adult	Depression	CBT	12 weeks	Weekly	Treatment	CMHC	-

	(2024) / Taiwan		21.4 (1.0)	awareness and resourcing media				as usual	(suicidal ideation) TDICS (Depression)	
14	Liu (2023) / China	RCT	Adult 17.7 (1.6)	loving-kindness meditation	Other (Medication)	8 weeks	Weekly (3 times a week)	waitinglist	BSSI (suicidal ideation)	-
15	Nagamitsu (2022) / Japan	RCT	Adolescent -	Mugimaru	CBT	4 weeks	Weekly	waitinglist	DSRS-C (Depression)	16 weeks
16	Nicol (2022) / USA	RCT	Adolescent 14.7 (1.7)	W-GenZ	CBT	12 weeks	Daily	waitinglist	PHQ-9 (Depression) GAD-7 (Anxiety)	12 weeks
17	Rodante (2020) / Argentina	RCT	Adult 29.9 (6.8)	CALMA	3th wave (DBT)	4 weeks	Weekly	Treatment as usual	SITBI (suicidal ideation)	-
18	Soltani (2024) / Iran	RCT	Adult -	Yara	N/R	12 weeks	Daily	Treatment as usual	BSSI (suicidal ideation) STAI (Anxiety)	-
19	Stallard (2024) / UK	RCT	Adolescent 15.6 (1.4)	BlueIce	CBT	12 weeks	Whenever needed	Treatment as usual	MFQ (Depression) RCADS (Anxiety)	24 weeks
20	Torok (2022) / Australia	RCT	Adult 21.5 (2.2)	LifeBuoy	3th wave (DBT, ACT)	6 weeks	Whenever needed	attention control app	SIDAS (suicidal ideation) PHQ-9 (Depression) GAD-7 (Anxiety)	12 weeks
21	Torok (2025) / Australia	RCT	Adult 19.9 (2.5)	LifeBuoy	CBT	8 weeks	Weekly	attention control app	SIDAS (suicidal ideation) PHQ-9 (Depression) GAD-7 (Anxiety)	16 weeks
22	Winslow (2022) / USA	RCT	Adult 37.4 (7.7)	-	CBT	12 weeks	Whenever needed	Treatment as usual	DASS (Depression, Anxiety)	-

Notes. RCT = Randomized Controlled Trial; NRS = Non Randomized Study; CBT = Cognitive Behavioral Therapy; DBT = Dialectical Behavior Therapy; ACT = Acceptance and Commitment Therapy; MI = Motivational Interviewing.
